# Comparing Repetition Priming Effects in Words and Arithmetic Equations: Robust Priming Regardless of Color or Response Hand Change

**DOI:** 10.3389/fpsyg.2017.02326

**Published:** 2018-01-10

**Authors:** Ailsa Humphries, Zhe Chen, Ewald Neumann

**Affiliations:** Department of Psychology, University of Canterbury, Christchurch, New Zealand

**Keywords:** repetition priming, arithmetic, words, stimulus–response binding, facilitation of component processes

## Abstract

Previous studies have shown that stimulus repetition can lead to reliable behavioral improvements. Although this repetition priming (RP) effect has been reported in a number of paradigms using a variety of stimuli including words, objects, and faces, only a few studies have investigated mathematical cognition involving arithmetic computation, and no prior research has directly compared RP effects in a linguistic task with an arithmetic task. In two experiments, we used a within-subjects design to investigate and compare the magnitude of RP, and the effects of changing the color or the response hand for repeated, otherwise identical, stimuli in a word and an arithmetic categorization task. The results show that the magnitude of RP was comparable between the two tasks and that changing the color or the response hand had a negligible effect on priming in either task. These results extended previous findings in mathematical cognition. They also indicate that priming does not vary with stimulus domain. The implications of the results were discussed with reference to both facilitation of component processes and episodic memory retrieval of stimulus–response binding.

## Introduction

Repetition priming (RP) refers to improved processing of a stimulus when that stimulus, or a similar one, is repeated compared to when it appears the first time. There are two commonly used paradigms: the prime-probe couplet paradigm, in which a prime, whose response may or may not be required, is followed almost immediately by a probe (e.g., Dehaene et al., 1998; Reynvoet et al., 2002; Kunde et al., 2003); and the study-test block paradigm, in which both the study and test blocks consist of a series of trials, and a response is required on every trial (e.g., Dobbins et al., 2004; Horner and Henson, 2009, 2011; Salimpoor et al., 2010). In both paradigms, the relationship between the prime and probe or between items in the study and test blocks is manipulated. The manipulation can concern various aspects of a stimulus, including identity (e.g., the same stimulus is repeated vs. a new stimulus is used), representational form (e.g., a numeral presented in the same notation vs. in different notations such as changing from an Arabic digit to a word form), classification (e.g., a stimulus classified in the same way vs. in different ways such as changing from “bigger than a shoebox” to “smaller than a shoebox”), and response (e.g., using the same hand to respond vs. switching to a different hand to respond). Although the magnitude of RP can be influenced by the similarity between the primed/studied items and the unprimed/new items, there is evidence that RP is remarkably robust, indicating that RP can occur at multiple levels of abstraction depending on task demand (see Henson et al., 2014, for a review). The presence of RP shows that behavior is not just driven by the current stimulus situation but is also affected by what has been processed in previous stimulus situations (Schacter, 1990).

There are two main theoretical perspectives that account for RP. The first perspective emphasizes the role of facilitation of component processes that give rise to a response when a stimulus is repeated. The facilitation can be due to changes in neural responses related to stimulus, task, and/or response features, enabling more efficient processing when the same or a similar stimulus is subsequently presented (see Grill-Spector et al., 2006, for a review). The second perspective emphasizes direct binding between a stimulus and response during encoding, and the role of episodic memory during retrieval. RP is explained as the result of a match (or mismatch) between a retrieving cue upon perceiving a stimulus and the context in which that same stimulus, or a similar one, was encountered previously. Theories belonging to this perspective include the instance theory (Logan, 1988, 1990), the event file theory (Hommel, 1998, 2004), and the action-trigger theory (Kunde et al., 2003), among others. Although their details differ, these theories all recognize, to various degrees, multiple levels of binding (e.g., binding among various stimulus features, locations, and the actions performed on the stimulus).

Most previous studies on RP used stimuli such as words, objects, and/or faces (e.g., Logan, 1988; Dobbins et al., 2004; Horner and Henson, 2009, 2011, 2012; Valt et al., 2015). Not many studies used numbers as stimuli (e.g., Dehaene et al., 1998; Naccache and Dehaene, 2001; Reynvoet et al., 2002; Kunde et al., 2003), and even fewer required participants to perform arithmetic computation on the numbers (e.g., Sciama et al., 1999; Salimpoor et al., 2010; Scheepers et al., 2011; Scheepers and Sturt, 2014). As the study of RP can reveal the underlying learning mechanisms that give rise to the processing efficiencies gained from stimulus repetition, it is important to know whether the mechanisms that underlie RP with conceptually rich stimuli such as words and objects are the same as those that underlie mathematical cognition. This question is particularly important for the contemporary society because of the growing reliance on technology and the introduction of computer coding as a core curriculum area within primary school education in many countries.

Although abstract numerical information and the more conceptually elaborative linguistic information appear to be represented in similar associative networks (Ashcraft and Battaglia, 1978; Ashcraft and Stazyk, 1981) and rely on a common underlying syntactic structure (Scheepers et al., 2011; Scheepers and Sturt, 2014), to date, no direct comparison has been undertaken in terms of whether RP effects are also equivalent in tasks that require semantic categorization of words and arithmetic categorization of equations. Furthermore, whereas long-lag RP has been reported in a number of word tasks (e.g., Scarborough et al., 1977; Bentin and Moscovitch, 1988; Kersteen-Tucker, 1991), so far, only one study has explored long-lag RP (an average lag of 16 trials) in a math task (Salimpoor et al., 2010). In that study, participants performed a three-operand arithmetic verification task in some blocks (e.g., to determine whether “3 + 5 - 4 = 4” is correct or incorrect) and a visual search task in the other blocks (e.g., to judge whether “4 @ 3 & 2 o 5” contained the digit ‘5’ or not) in a study-test block design, and significant RP was found in response latencies in both tasks. As long-lag RP in mathematical cognition has not been studied very much, it is important to find out whether the results reported in Salimpoor et al. (2010) could be generalized to a similar task.

In the two experiments reported below, we used a within-subjects design to compare the magnitude of RP in both a word and a math task. We manipulated stimulus identity and color in Experiment 1, and motor action in Experiment 2. These experiments were designed with the following objectives in mind: (1) to compare the magnitude of RP between a word and a math task, and to determine whether RP would be affected to a similar degree in the two tasks by a change in a task irrelevant feature dimension (Experiment 1) or by a change in motor action (Experiment 2); and (2) to investigate whether RP in a two-operand arithmetic categorization task could survive an average of 15 intervening trials.

An important feature in most previous studies on RP is the use of an *absolute*-*differences* method of analysis in calculating the results (e.g., Sciama et al., 1999; Horner and Henson, 2009). In this method, the data from the prime trials or study blocks are not included in the analysis of RP. Instead, only those data from the probe trials or test blocks are analyzed. Thus, RP is based on comparing the *absolute difference* in RT and accuracy between the experimental (i.e., the primed or studied) condition and the control (i.e., the non-primed or studied) condition. This method of analysis fits with the conception on RP as a phenomenon where studied (primed) items are more efficiently and effectively responded to than non-studied (un-primed) items (Schacter, 1990). However, given that theories of RP attempt to explain current processing as a result of prior processing, it would seem important that all characteristics of the initial state be taken into consideration and, where possible, analysis be based on the *relative difference* in RT and accuracy from study and test trials. For example, in a study-test block design, instead of directly comparing the performance in the studied test block with that in the unstudied test block (i.e., the absolute difference method), one first calculates two difference scores: one between the study and test blocks for the studied condition, and the other between the study and test blocks for the unstudied condition. Thus, whereas the absolute difference method is built on the assumption that performance does not differ across conditions in the study blocks, the relative difference method takes into account (empirically) performance in these blocks. As variations across conditions during study are more likely to be captured in the relative difference method rather than in the absolute difference method as suggested by recent findings of anti-priming (e.g., Marsolek et al., 2006; Zhang et al., 2017), the relative difference method provides a more complete picture of RP. In the experiments reported here, we used this method in our data analyses.

## Experiment 1

Experiment 1 investigated whether RP could be found in a blocked study-test design with an average lag of 15 intervening trials in both a word and arithmetic categorisation task, and whether changes in color, a task irrelevant feature, would affect the magnitude of RP to a similar degree in the two tasks.

Previous research on memory has shown that performance is influenced by the match in mental operations required of the task at study and the task at test. A task can be classified as being primarily conceptually driven or data driven (Blaxton, 1989). A conceptually driven task (or conceptual task) requires the processing of the semantic information of a stimulus. In contrast, a data driven task (or perceptual task) requires the processing of the perceptual features of a stimulus. According to the principle of transfer-appropriate processing (Morris et al., 1977; Blaxton, 1989; Roediger et al., 1989), memory performance is improved to the extent the mental operations performed at study overlap with those performed at test. Consistent with this principle, RP is typically found when the type of processing involved at study is evoked at test (e.g., Blaxton, 1989; Mulligan, 1996; Franks et al., 2000; Spataro et al., 2011). Furthermore, when a specific type of processing (e.g., the processing of perceptual features) is not required at test, changes in stimulus features relating to that type of processing between study and test do not affect the magnitude of priming (Blaxton, 1989).

In Experiment 1, in both the study and test blocks, participants categorized a word (e.g., tiger) as referring to an animal or an object in the word task, and they determined whether the answer to an arithmetic equation (e.g., 5 + 12) was an odd or an even number in the math task. As both tasks were conceptual tasks that relied primarily on the processing of semantic information, a change in the ink color of a stimulus, a task irrelevant perceptual feature, from study to test should not influence RP very much so long as the identity of the stimulus remained the same between study and test.

While it was unknown how a change in study might affect priming in test in a linguistic task relative to an arithmetic task, there is evidence for structural priming between the two domains (e.g., Scheepers et al., 2011; Scheepers and Sturt, 2014). Scheepers and Sturt (2014, Experiment 1) used a prime-probe couplet paradigm, with the prime being an arithmetic equation that had a left- or right-branching structure (e.g., 3 × 4 + 6 vs. 3 + 4 × 6), and the probe a linguistic expression that also had a left- or right-branching structure (e.g., alien monster movie vs. lengthy monster movie). The task was to solve the equation and then to rate the sensicality, on a 1–5 scale, of the linguistic expression. A robust priming effect was found. Participants rated the linguistic expressions as being more sensical when the prime and probe had the same structure compared with when the two had different structures. In a subsequent experiment, structural priming was again found when the prime was a linguistic expression and the probe was an arithmetic equation. These results suggest that arithmetic and language share syntactic representations. They also raise the possibility that if we manipulate an aspect of a stimulus such as color between study and test in both a word task and a math task, the effect on priming might be comparable between the two tasks.

### Method

#### Ethics Statement

The study reported here received prior ethical approval from The University of Canterbury Human Ethics Committee. The committee approved the consent form and experimental procedure. Written consent was obtained from the participants. All subjects gave written informed consent in accordance with the Declaration of Helsinki.

#### Participants

Thirty-one participants were recruited from the University of Canterbury (9 males and 22 females) between the ages of 17 and 44 years (*M* = 20.8 years, *SD* = 5.7 years). All the participants were enrolled in a first year psychology course and participated in return for course credit.

#### Apparatus and Stimuli

The experiments were presented on a PC with a 50-cm × 30-cm monitor in width and height. E-Prime 2.0 with a refresh rate of 60 ms was used to generate the stimuli and to collect responses. Participants were tested individually in a dimly lit room. The viewing distance was approximately 60 cm.

Each trial consisted of a central fixation followed by a word or an arithmetic equation presented at the center of the screen. The fixation was a black cross that extended 0.06 degrees of visual angle in both length and width. Both the word and the arithmetic equation were written in Courier New, font size 40. Depending on the experiment condition, the stimuli were either black or colored. In the latter condition, the colors used were black, blue, cyan, green, lime, magenta, maroon, navy, olive, orange, purple, and red.

In the word task, which required participants to determine whether the word referred to an animal or an object, the stimulus set consisted of 184 words that varied in length from three to nine letters.^1^ Half of them referred to an animal and half to a household object. In the math task, which required participants to determine whether the answer to the equation was an odd or an even number, the stimulus set consisted of 216 equations. All equations were in the format of a single digit number plus a two-digit number, with half of them starting with the single digit number (e.g., 5 + 15) and the other half starting with the double-digit number (e.g., 16 + 4). Half of the equations had an odd answer and half an even answer. Within these constraints, in both the word and the math task, stimuli were randomly selected to each condition for each participant.

#### Design and Procedure

The experiment used a 2 (task: word vs. math) × 2 (feature: ID vs. color) × 2 (condition: same vs. change) within-subjects design. Participants completed both tasks in one sitting and each task included two sessions: an ID session, in which the identity of the stimulus was manipulated (ID-same vs. ID-change), and a color session, in which the color of the stimulus was manipulated (color-same vs. color-change). Only one stimulus dimension varied in each session. In other words, all stimuli had the same color in the ID session and all stimuli had the same identity in the color session. In both sessions, only stimulus identity was task relevant while color was task irrelevant. The order of the two tasks and the two sessions was counterbalanced across participants.

Both tasks followed the same procedure, and within each task the two sessions followed the same blocked design with four rounds of study-test cycles. Each of these four cycles consisted of two mini-blocks of eight study words/equations followed by two mini-blocks of eight test words/equations. This gave a total of 16 mini-blocks (8 study mini-blocks plus 8 test mini-blocks), or 32 trials per study-test cycle and 128 trials per session, for a total of 256 trials in each task.

In the ID session, all stimuli were black. In the ID-same condition, the words or equations used in the study phase were re-used in the test phase. In the ID-change condition, different words or equations were used in the study and test phases. No words or equations were used twice within a task except to fulfill the repetition condition. In addition, the set of words or equations used in the ID session was not re-used in the color session, so there was no overlap of stimuli between the two sessions.

**FIGURE 1:**
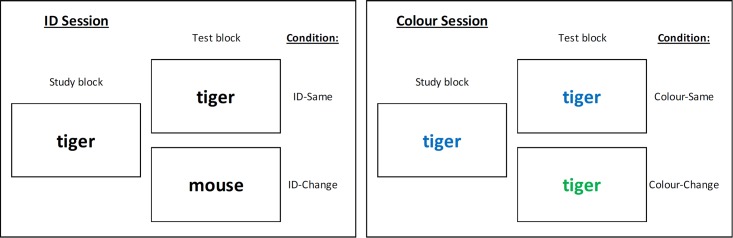
A schematic representation for the ID and color sessions in the word classification task in Experiment 1. The allocation of words to condition and the order in which the words were presented within each of the study and test blocks was randomized. Note that the identity of the stimuli in the color session was always the same between the study and test blocks regardless of whether there was a change in color between the two blocks.

In the color session, all words or equations were presented in colored font and the identity of the words or equations in the study block was always repeated in the test block. In the color-same condition (8 mini-blocks in total, with 4 mini-blocks in the study phase and 4 in the test phase), the stimuli had the same color in both the study phase and the test phase. This resulted in the use of four different colors, one for each study-test cycle. In the color-different condition (8 mini-blocks in total, with 4 mini-blocks in the study phase and 4 in the test phase), the stimuli had one color in the study phase but a different color in the test phase. This led to the use of eight different colors, with a unique color in every mini-block. No colors were used twice between any two mini-blocks within a task except to fulfill the repetition condition. Within each study-test cycle, the order of presentation of individual stimuli in a mini-block was always randomized and the order of the two mini-blocks at test was also randomized. Hence, the maximum possible lag between presentations of the same stimuli was 30 trials and the minimum was zero, with an average lag of 15 intervening trials. **Figure 1** shows the four conditions as they relate to the study and test cycles in both the ID and color sessions for the word task. The conditions were the same for the math task.

Each trial began with a fixation cross for 1,000 ms, followed by a target display for 120 ms, and then a blank screen until a response was made. The inter-trial stimulus interval was 1,000 ms. The participants used the index and middle fingers of their right hand to press one of the two labeled keys on a computer keyboard. In the word task, the participants pressed the “o” key if the word referred to an animal and the “p” key if the word referred to an object. In the math task, they pressed the “o” key if the answer to the equation was an odd number and the “p” key if it was an even number.

Each participant first completed a brief practice block of 32 trials for each task before proceeding to the experiment proper. None of the stimuli used in the practice block were used in the experimental blocks. All participants were encouraged to take a break between tasks and between the two sessions within each task. The total amount of time for the experiment was approximately 40 min.

### Results and Discussion

Data exceeding 3 SD (both above and below) from each individual participant’s mean RT were excluded. This resulted in the exclusion of less than 2% of the data. Data from five participants were also excluded due to an error rate exceeding 25% in one or more conditions, and data from three further participants were excluded due to their median RT being more than 3 SD above the average of the median RTs for all participants.

The means of median RTs and error rates for the remaining participants are shown in **Table 1** for the word task and **Table 2** for the math task. For each participant, we calculated the difference score between the study and test blocks (Study – Test) for each condition, and these difference scores were then used to compute RP. Hence, the statistical analyses and the interpretation of the results reported below are based on the data using the relative-difference method of analysis.

**Table 1:** Means of median reaction times, expressed in milliseconds (ms), and percentage of errors (%) for the classification of words in Experiment 1.

	Reaction time (ms)				Percentage error (%)			
	Study		Test		Study		Test	
	*M*	*SD*	*M*	*SD*	*M*	*SD*	*M*	*SD*
ID-same	619	77	584	58	3.2	5.1	3.5	4.1
ID-change	620	81	625	77	3.7	4.6	5.1	6.7
Color-same	614	63	584	62	3.5	3.5	3.2	3.8
Color-change	632	81	592	56	5.1	5.2	4.9	5.2

**Table 2:** Means of median reaction times, expressed in milliseconds (ms), and percentage of errors (%) for the classification of equations in Experiment 1.

	Reaction time (ms)				Percentage error (%)			
	Study		Test		Study		Test	
	*M*	*SD*	*M*	*SD*	*M*	*SD*	*M*	*SD*
ID-same	1,186	211	1,093	233	8.5	5.9	6.8	5.9
ID-change	1,209	241	1,205	276	9.6	6.9	7.8	5.1
Color-same	1,135	259	1,095	264	8.5	6.9	7.7	6.4
Color-change	1,142	251	1,081	255	9.4	6.5	4.8	3.6

**FIGURE 2:**
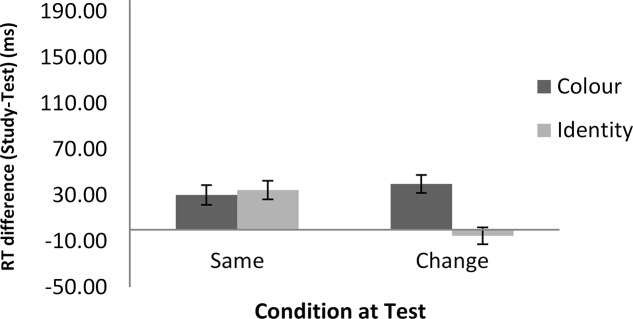
RT differences between the study and test blocks in the word classification task as a function of feature and condition in Experiment 1. Error bars show ±1 standard deviation of the mean. A positive number indicates faster RT in the test block than in the study block. A negative number indicates slower RT in the test block than in the study block.

**FIGURE 3:**
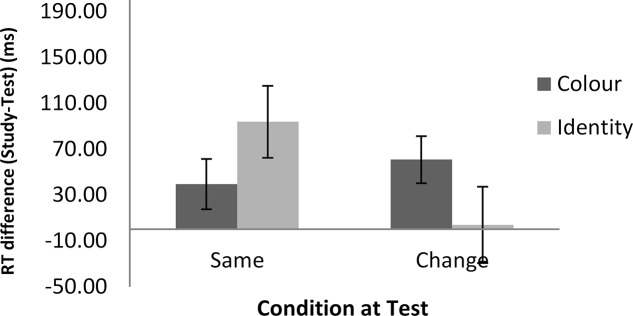
RT differences between the study and test blocks in the math task as a function of feature and condition in Experiment 1. Error bars show ±1 standard deviation of the mean.

**Figures 2**, **3** show the mean of the difference score in RT in each condition for the word and the math task, respectively. A 2 (task: word vs. math) × 2 (feature: ID vs. color) × 2 (condition: same vs. change) repeated-measures analysis of variance (ANOVA) was conducted on the RT data (i.e., the difference scores between the study and the test blocks). The results showed that the intercept was significantly different from zero, *F*(1,22) = 32.87, *MSE* = 7703*, p* < 0.001, $\eta_{p}^{2}$ = 0.60. As we used difference scores in the analysis, this result indicated that RTs were faster at test than at study. There was also a main effect of task, *F*(1,22) = 4.51, *MSE* = 6148, *p* < 0.05, $\eta_{p}^{2}$ = 0.17, suggesting a larger RT improvement in the math mask (49 ms) than in the word task (25 ms). In addition, feature and condition interacted, *F*(1,22) = 8.96, *MSE* = 8268, *p* < 0.01, $\eta_{p}^{2}$ = 0.29. For the ID session, the magnitude of difference in RT between the study and test blocks was significantly larger in the ID-same condition (64 ms) than in the ID-different condition (-1 ms), indicating RP. For the color session, there was no significant difference in priming between the color-same condition (35 ms) and the color-different condition (50 ms). The latter result indicates no reduction in priming regardless of whether there was a color change from the study to the test block. No other effects reached significance.

A similar ANOVA was conducted on the error rates. The intercept was again significantly different from zero, *F*(1,22) = 5.74, *MSE* = 30*, p* < 0.05, $\eta_{p}^{2}$ = 0.21, indicating more accurate responses at test than at study. The main effect of task was significant, *F*(1,22) = 17.81, *MSE* = 16*, p* < 0.001, $\eta_{p}^{2}$ = 0.45, suggesting that the participants made more improvement between the study and test blocks in the math task (2.2%) than in the word task (-0.3%). No other effects were significant.

The most important finding in Experiment 1 is the remarkable similarity in the pattern of data between the word and math tasks. In both tasks, a significant RP effect was found in the ID session, indicating that RP can survive an average lag of 15 intervening trials in both tasks. The finding of the RP in the math task extended the results of Salimpoor et al. (2010), who used a three-operand arithmetic verification task and found significant long-lag RP that survived an average of 16 intervening trials.

The improvement in RT from the study to the test block may appear to be larger in the math task (49 ms with an error rate of 2.2%) than in the word task (25 ms with an error rate of 0.3%). However, the difference in RT was largely due to the math task taking substantially longer to complete (mean RT = 1,143 ms) than the word task (mean RT = 609 ms). In terms of percentage change from study to test, which is a more accurate measure for RP in the present paradigm, the improvement in performance was 4% in both tasks, indicating that the magnitude of RP was comparable in the two tasks. With regard to the difference in error rates, the overall error rate in the word task (4%) was lower than that in the math task (7.9%). The comparable performance between the study and test blocks in the word task could therefore be caused by a ceiling effect. There was not much room for performance to improve from study to test. Taken together, these results showed very little difference in the magnitude of priming between the word and math tasks.

Color change had no discernible effect on the magnitude of priming in either the word or the math task. These results are consistent with prior research showing that RP arises primarily from task relevant features (Maljkovic and Nakayama, 1996; Hillstrom, 2000; but see Huang et al., 2004). They are also in line with the findings of several previous memory studies that involved a color change in stimuli from study to test (e.g., Szymanski and MacLeod, 1996; MacDonald and MacLeod, 1998; Newell et al., 2008; but see Stone et al., 2000). Szymanski and MacLeod (1996) showed their participants words printed in different colors in the study phase. In one block (the reading condition), the participants read each word aloud (a task that evokes both perceptual and conceptual processing). In the other block (the color naming block), they named the ink color of each word (a task that evokes primarily perceptual processing). In the test block, in which all stimuli were printed in white, half the participants completed a memory recognition task while the other half a lexical decision task. For the memory recognition task, which is a task that relies heavily on conceptual processing, performance was better for the words that had appeared in the reading condition compared with the color naming condition. However, for the lexical decision task, which is a task that evokes predominantly perceptual processing because it can be performed on the basis of overall lexical familiarity without complete lexical identification, at least for common words (Grainger and Jacobs, 1996), performance was comparable for words that had appeared in the reading and the color naming conditions. These and similar results reported by other researchers (e.g., MacDonald and MacLeod, 1998; Newell et al., 2008) are consistent with the findings of Experiment 1 in the present study. Together, they indicate that a change in stimulus color from study to test do not necessarily affect the magnitude of priming so long as color is not a task relevant attribute at test.

## Experiment 2

Experiment 1 showed no reduction in RP in either the word or the math task when there was a color change between the study and the test. In Experiment 2, we investigated the effect of motor action on RP in the two tasks. Instead of manipulating ID or color, we manipulated the response hand so that participants either used the same hand to press the response keys in both the study and test blocks, or switched hand between the study and the test block. The goal of the experiment was to determine whether a change in response hand would affect RP in the math task, and if so, whether the magnitude of the effect would again be similar between the word and math tasks.

### Method

#### Participants

Twenty-seven new participants from the same participant pool were recruited from the University of Canterbury (5 males and 22 females) between the ages of 17 and 59 years (*M* = 21 years, *SD* = 8.3 years) in return for either course credit or a $10 voucher.

#### Apparatus and Stimuli

Both the apparatus and stimuli were the same in Experiment 2 as those in Experiment 1 except for the following three differences. First, the test blocks always contained the same words or equations as the study blocks, and all stimuli were presented in black. Thus, unlike Experiment 1, there was no change in identity or color between the study and test blocks. As in the previous experiment, the stimuli on each trial were selected in a random order. Second, each study-test cycle consisted of one mini-block of eight study items followed by one mini-block of eight test items. This resulted in a maximum possible lag between repeated stimuli being 14 trials and the minimum being zero, with an average lag of 7 intervening trials. We reduced the length of the intervening trials to increase the sensitivity of the experiment, as there is evidence that priming effects are stronger with reduced lags (Henson et al., 2004). Third, each mini-block was preceded by an instruction display, which informed the participants which hand (left or right) they should use to perform the task in the subsequent trials. If the correct hand to use was the left hand, they had to press either the “w” or the “e” key (the response keys designated for left hand responses) for the next mini-block to start. If the correct hand to use was the right hand, they had to press either the “o” or the “p” key (the response keys designated for right hand responses) to proceed. In total, each participant was presented with eight ‘hand-same’ study-test cycles interwoven with eight ‘hand-change’ study-test cycles. In the ‘hand-same’ cycle, the same hand was used at study and test (four left hand and four right hand cycles). In the ‘hand-change’ cycle, the response hand was changed from study to test (four left to right and four right to left cycles). These four combinations were randomized and presented an equal number of times to each participant.

#### Design and Procedure

The experiment used a 2 (task: word vs. math) × 2 (condition: same vs. change) within-subjects design. The order of the task was counterbalanced, with half the participants completing the word task first, and the other half completing the math task first.

As in Experiment 1, each trial began with a fixation display for 1,000 ms followed by the task display for 120 ms, and then a blank screen until response. After every eight trials, based on instruction, the participants either used the same hand to respond or switched to a different hand. For right hand responses, the participants pressed the same keys as in Experiment 1, i.e., the “o” key for “odd” or “animal,” and the “p” key for “even” or “object.” For left hand responses, they pressed the “w” key for “even” or “object,” and the “e” key for “odd” or “animal.” Before the start of the experiment, the participants were instructed to place both hands on the keyboard, with the relevant fingers resting on the four response keys. All the other aspects of the procedure were the same as those in Experiment 1. The entire experiment took about 45 min to complete.

### Results and Discussion

The data were treated in the same way as that in Experiment 1, and this excluded less than 2% of the data. Five participants’ data were not included in further analyses, three due to high error rates (exceeding 25% in one or more conditions) and two due to long RTs (more than 3 SD above the average of the median RTs for all participants).

**FIGURE 4:**
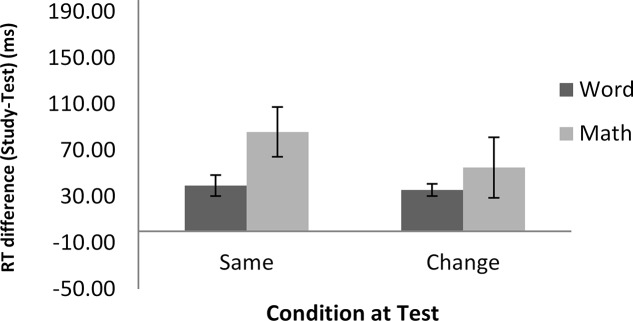
RT differences between the study and test blocks as a function of condition in Experiment 2. Error bars show ±1 standard deviation of the mean.

**Table 3** shows the means of median RTs and error rates in each condition. As in Experiment 1, we calculated the difference scores between the study and test blocks, and **Figure 4** shows the means of the difference scores in RTs.

**Table 3:** Means of median reaction times, expressed in milliseconds (ms), and percentage of errors (%) for the classification of words and equations in Experiment 2.

	Reaction time (ms)				Percentage error (%)			
	Study		Test		Study		Test	
	*M*	*SD*	*M*	*SD*	*M*	*SD*	*M*	*SD*
Word hand-same	630	84	590	64	3.9	3.2	3.1	3.6
Word hand-change	626	76	591	67	4.8	5.4	3.8	4.7
Math hand-same	1,245	312	1,159	273	9.6	6.4	7.8	4.9
Math hand-change	1,213	302	1,158	254	9.3	6.1	9.2	5.7

Two 2 (task: math vs. word) × 2 (condition: same vs. change) repeated-measures ANOVAs were conducted, one on the error rates and the other on the RTs, again using the difference scores between the study and the test blocks. The intercept was significantly different from zero in both accuracy, *F*(1,21) = 4.94, *MSE* = 15, *p* < 0.05, $\eta_{p}^{2}$ = 0.19, and RTs, *F*(1,21) = 28.46, *MSE* = 9020, *p* < 0.001, $\eta_{p}^{2}$ = 0.57, indicating more accurate and faster performance at test than at study. The main effect of task in RT was marginally significant, *F*(1,21) = 4.09, *MSE* = 5965, *p* = 0.06, $\eta_{p}^{2}$ = 0.16, suggesting a larger difference between the study and test blocks in the math task (70 ms) than in the word task (38 ms). As in Experiment 1, this difference was due to longer RTs in the math task (1,184 ms) than in the word task (609 ms). In terms of percentage change from study to test, this equated to a 6% improvement in RT in each task. No effect of task was found in the error rates. Importantly, there was no significant main effect of condition, or task by condition interaction, in either error rates or RTs. These results indicate that a change in hand between the study and test blocks did not reduce the magnitude of RP in either the word or the math task.

Once again, a similar pattern of data was found between the word and math tasks. Participants showed significant priming in both tasks, and a change in response hand did not affect the magnitude of priming in either task. These results provided converging evidence to the finding of Experiment 1, which also showed comparable performance between the word and math tasks.

The absence of a response congruency effect in motor action in the word task may appear to be inconsistent with previous studies that showed a reduction in priming when a change in motor action occurred. Using a study-test block design, Horner and Henson (2009, Experiment 6) showed participants pictures of objects, and the task in the test block was to respond whether the object was bigger than a shoebox by pressing one of two response keys for a “Yes” or “No” answer. In the study block, there were three conditions: the same action/same decision condition, in which both the action (i.e., keypress responses) and the decision (i.e., Yes/No answer) were the same as those in the test block; the different action/same decision condition, in which the same Yes/No task as in the test block was performed with verbal responses; and the different action/different decision condition, in which the task was to name the target object verbally. Compared to new stimuli, priming was significantly larger in the same action/same decision condition than in the different action/same decision condition, which in turn was larger than in the different action/different decision condition. Thus, priming was reduced when there was a change in motor action or a change in decision (see also Dobbins et al., 2004, for a related finding).

There are many methodological differences between Horner and Henson’s (2009) experiment and Experiment 2 in the present study. These differences include the type of stimuli (objects vs. words), response decision (yes/no vs. animal/object), presentation duration (2,000 ms vs. 120 ms), motor action (verbal/keypress vs. keypress only), and the method of data analysis (absolute difference vs. relative difference), among others. Although the exact cause for the difference in results is unclear, a possible candidate that might contribute to the different results between the two experiments could concern the manipulation of motor action, which was between verbal and keypress responses in Horner and Henson (2009), but between left and right hand responses in the present experiment. It is possible that our manipulation of response hand was not sufficiently sensitive to elicit the effect of a change in motor action on RP between study and test. It is conceivable that a change in motor action requires more attentional resources when the change is large (e.g., from a hand response to a verbal response) than when it is small (e.g., from a left hand response to a right hand response). As attention strengthens binding (Logan, 1990; Treisman, 1992), the S–R binding should be stronger in the former case than in the latter case. Consequently, all else being equal, a response congruency effect is more likely to manifest in RP when the change in motor action is between different response modalities as in Horner and Henson’s (2009) study than when the change is within the same response modality as in the present experiment.

## General Discussion

The main goal of this study was to investigate and compare the effects on behavioral priming of altering the color or the response hand for repeated, otherwise identical, stimuli in a word and a math task using a within-subjects design. Using a novel arithmetic categorization task, our results indicate that a single, brief, task relevant experience with an arithmetic equation affects subsequent performance over an average lag of 15 intervening trials. This result extends previous findings (e.g., Salimpoor et al., 2010) to a new computationally challenging task within the domain of mathematical cognition.

No previous studies had used a within-subjects design to directly compare the magnitude of RP in a predominantly linguistic task with a task that requires arithmetic computation. Using a within-subjects study-test block design, we asked participants to complete both a word and a math task. Our results show that the magnitude of RP was comparable between the two types of task, and that changing a task irrelevant object feature such as color or the hand to respond had a negligible effect on RP in either task.

The lack of a color congruency effect in Experiment 1 can be explained in one of several ways. First, when a task at test required only conceptual processing, priming occurred at a semantic level rather than at the level of perceptual features. This indicates that priming operates at the level of processing at which the task is directed (Schacter, 1990), a result consistent with the principle of transfer-appropriate processing (Morris et al., 1977; Blaxton, 1989; Roediger et al., 1989). Thus, for a perceptual discrimination task, perceptual features will be primed, while for a conceptual categorisation task, semantic features will be primed (Jacoby, 1983; Kirsner et al., 1986; Schacter, 1990). Second, color did not bind with the identity of the stimulus because it was task irrelevant (Hommel, 1998). The sense that color was irrelevant was likely to be enhanced by the use of 12 different color values in Experiment 1, and this led to the observed absence of a perceptual congruency effect in color. It has been suggested that binary manipulations elicit a mutual inhibition effect, where the activation of one feature serves to inhibit the activation associated with the opposing feature (Hommel, 2009). According to this account, the use of two colors (i.e., red and green) creates a competitive interaction where repeating a word in red results in a behavioral advantage from both the facilitation of red and the inhibition of green, and this interaction is conducive to the manifestation of RP. In the present study, such a mutual inhibition effect was not elicited, as there were a dozen different color values.

It is also possible that color and identity did not bind because color was simply ignored. This is likely because a single color was mapped to multiple stimulus identities in our study, and this one-to-many mapping might have encouraged participants not to attend to color. As the strength with which features are bound depends on attention (Logan, 1990; Treisman, 1992), an ignored feature may be precluded from the binding process, especially when stimulus displays are presented very briefly, which was the case (120 ms of target presentation duration) in Experiment 1. Finally, color did bind but was not available upon retrieval. Whereas the identity of stimuli was retained over multiple intervening trials, the delay may have been too long for the binding of color and identity to survive, in line with the claim that a delay in the repetition of an S–R pairing can lead to decay in the strength of binding (Hommel, 1998).^2^ In the present study, the decay may start quite early, partly due to the use of multiple colors, and partly to the one-to-many mapping between color and stimulus identities. Both factors would weaken the strength of the binding if binding between color and stimulus identity occurred. Although each of the above interpretations can account for the absence of the perceptual congruency effect in Experiment 1, it is likely that the observed result was caused by more than one of the reasons stated above.

Previous research using numbers as stimuli has shown that changing task irrelevant features can affect the magnitude of priming in some situations but not in others. Naccache and Dehaene (2001) reported no reduction in priming in a masked prime-probe number categorisation task (probe smaller or larger than “5”) regardless of whether the prime and probe matched in notation (Arabic digit vs. word). Using a study-test block design that required participants to perform a two-operand arithmetic task, Sciama et al. (1999) found attenuated priming in the notation change condition compared to the notation same condition when the numerals in the test blocks were presented in atypical notations such as in words or dot configurations. These results were attributed to atypical notations requiring additional attentional resources for arithmetic tasks, and the demand of attention in turn enhanced ‘form-specific’ associations. In the present study, we used Arabic digits in both the study and test blocks. Our results are thus generally consistent with previous studies.

The factors we discussed in relation to color change in Experiment 1 can also apply to the results concerning hand change in Experiment 2. One may wonder whether response hand can be considered as a task irrelevant feature in Experiment 2. After all, the use of the correct response hand was an important requirement in the experiment. That being said, it is important to remember that the task in Experiment 2 was still about the semantic concept of the target stimuli, that the same hand was used within each mini-block of eight trials, that the participants had as much time as they needed to prepare the hand change before each mini-block, and most important, that the participants would no longer need to be concerned about which hand to use once a mini-block of trials started. This is because they would already be using the correct hand to respond at the beginning of any mini-block of trials as they first had to press one of the correct response keys designated for the correct hand (left or right) to terminate the response instruction display so that the experiment could proceed. Taking all of these into account, it seems reasonable to consider response hand as being a task irrelevant feature in Experiment 2, similar to color being a task irrelevant feature in Experiment 1. If this reasoning is correct, then the lack of a response congruency effect in Experiment 2 could be due to priming occurring at an abstract semantic level in accordance with the behavioral goal, the failure of binding between stimulus identity and motor action, and/or the decay of the binding from study to test.

The participants in both Experiments 1 and 2 showed comparable RP in the word and math tasks when a task irrelevant feature such as the color of a stimulus or the hand used to make a response changed from study to test. However, caution must be taken to generalize the present findings to other variables. As neither color nor the response hand change influenced the magnitude of RP in the present study, it is unclear whether comparable RP would still be found in a word and a math task if the variables manipulated had a significant effect on RP in one or both tasks. Future studies comparing the effects of RP in the two types of tasks should select a variable known to affect RP in a word and/or a math task. For example, to determine the effect of a perceptual feature on RP across the two tasks, instead of manipulating color, one can manipulate surface form, perhaps using a familiar font (e.g., Times New Roman) at study but an unusual font (e.g., [scale=.5]img001) at test. Similarly, to investigate the effect of motor action, instead of manipulating response hand, one can vary response modality (e.g., a hand response vs. a verbal response). As there is evidence that changes in surface form (Sciama et al., 1999) or response modality (Horner and Henson, 2009) can influence RP, one would expect a reduction in RP in the word and/or the math task. If the magnitude of reduction is similar in both tasks, this will provide converging evidence to the findings in the present study that the magnitude of RP is comparable in a predominantly linguistic task and in a task that requires arithmetic computation.

With regard to whether the RP observed in our experiments is best explained in terms of a facilitation account or a retrieval based S–R binding account, our results are equivocal. On the one hand, a facilitation account appears to be sufficient in accounting for the present results. The lack of a congruency effect in either Experiment 1 or Experiment 2 is consistent with the notion that RP in the present paradigm was driven by faster stimulus identification of repeated words or equations at a semantic level irrespective of task irrelevant object features or motor action. There was no evidence of an interaction between different stimulus features or between a stimulus feature and response. The improved performance for repeated stimuli can thus be the result of facilitated neural processing, which may manifest as an overall reduction in the amplitude of neuronal activation (Dragoi et al., 2002; Kohn and Movshon, 2003), a sharpening of response tuning in local networks of neurons (Wiggs and Martin, 1998; Freedman et al., 2006), more rapid onset of neural activation (James and Gauthier, 2006), and/or lowered activation threshold (Morton, 1979).

On the other hand, the lack of a response congruency effect cannot rule out an S–R binding account. As we discussed before, it was possible that binding between stimulus identity and motor action had occurred but did not survive the intervening trials. It was also possible that stimulus identity was simultaneously bound to multiple response codes. Horner and Henson (2009) identified three levels of response codes: the motor action level (e.g., left or right hand used in response), the decision level (e.g., a yes or no response), and the task-specific classification level (e.g., a bigger than or a smaller than question). They showed that each level can contribute to S–R binding. As we only manipulated motor action in our study, it was conceivable that the binding between identity and the other two levels of response code remained intact between the study and test blocks. Hence, no reduction in RP was found.

In summary, using a within-subjects design, we showed a remarkable similarity in the pattern of priming between a word and a math task, both when there was a color change and a response hand change. These results extended previous findings in mathematical cognition, and provided supporting evidence for the proposal that priming does not vary with stimulus domain (Ashcraft and Battaglia, 1978; Ashcraft and Stazyk, 1981) and that mathematical and linguistic information relies on a common representative structure (Scheepers et al., 2011; Scheepers and Sturt, 2014).

## Author Contributions

AH and ZC generated the experimental ideas. AH conducted the experiments, and analyzed the data with input from ZC. The manuscript was written by AH, revised by ZC. EN provided feedback on the manuscript, and proof-read it.

## Conflict of Interest Statement

The authors declare that the research was conducted in the absence of any commercial or financial relationships that could be construed as a potential conflict of interest.

## Footnotes

1 The words used in the present experiments were not taken from any published database, and no attempt was made to match the frequency of words in different experimental conditions. However, this should not influence the results of the experiments because the stimuli were randomly selected to each condition for each participant.

2 Unfortunately, we were unable to assess this statement empirically due to insufficient number of trials when the data were broken down into separate lags in the present study. In Experiment 1, each condition consisted of 32 trials. If we broke down the data into short (0–9 intervening trials), intermediate (10–19 intervening trials), and long (20–29 intervening trials) lags, each lag would only have an average of 10 trials after error trials were excluded. This prevented us from estimating participants’ performance as a function of lag in a meaningful way. A similar situation applies to the data in Experiment 2.
